# Early Critical Care Course in Children after Liver Transplant

**DOI:** 10.1155/2014/725748

**Published:** 2014-09-25

**Authors:** Vinay Kukreti, Hani Daoud, Sundeep S. Bola, Ram N. Singh, Paul Atkison, Alik Kornecki

**Affiliations:** ^1^Department of Pediatric Critical Care Medicine, Children's Hospital, London Health Sciences Centre, University of Western Ontario, 800 Commissioners Road East, P.O. Box 5010, London, ON, Canada N6A 5W9; ^2^Division of Respiratory Medicine, Hospital for Sick Children, University of Toronto, 555 University Avenue, Toronto, ON, Canada M5G 1X8; ^3^Department of Pediatrics, Children's Hospital, London Health Sciences Centre, University of Western Ontario, 800 Commissioners Road East, P.O. Box 5010, London, ON, Canada N6A 5W9; ^4^Children's Health Research Institute, 800 Commissioners Road East, P.O. Box 5010, London, ON, Canada N6A 5W9

## Abstract

*Objective.* To review the critical care course of children receiving orthotopic liver transplantation (OLT). *Methods.* A retrospective chart review of patients admitted to the pediatric critical care following OLT performed in our center between 1988 and 2011. *Results.* A total of 149 transplants in 145 patients with a median age of 2.7 (IQR 0.9–7) years were analyzed. Mortality in the first 28 days was 8%. The median length of stay (LOS) was 7 (4.0–12.0) days. The median length of mechanical ventilation (MV) was 3 (1.0–6.2) days. Open abdomen, age, and oxygenation index on the 2nd day predicted LOS. Open abdomen, age, amount of blood transfused during surgery, and PRISM III predicted length of MV. 28% of patients had infection and 24% developed acute rejection. In recent group (2000–2011) OLT was performed in younger patients; the risk of infection and acute rejection was reduced and patients required longer LOS and MV compared with old group (1988–1999). *Conclusion.* The postoperative course of children after OLT is associated with multiple complications. In recent years OLT was performed in younger children; living donors were more common; the rate of postoperative infection and suspected rejection was reduced significantly; however patients required longer MV and LOS in the PCCU.

## 1. Introduction

Over the past thirty years orthotopic liver transplantation (OLT) has become a standard procedure for end stage liver disease and certain metabolic diseases with a complex and challenging postoperative course [1]. Improvements in organ preservation, immunosuppression, and surgical techniques have improved survival and long term outcome of patients after OLT [2–4] however the early postoperative course remains challenging [5]. We conducted a retrospective chart review analysis of all patients who had undergone OLT in our center in order to report the clinical course after OLT and to capture potential changes that took place over the more recent years.

## 2. Materials and Methods

The study protocol was reviewed and approved by the Institutional Research Ethics Board of Western University, Ontario, Canada. Utilizing the PCCU, the pediatric liver transplant database and the hospital's medical records, a retrospective chart review study was performed. All patients aged 1 month to 18 years old who had undergone liver transplantation during the years 1988–2011 were identified. For each of the identified patients the demographic and clinical data (pre-, intra- and postoperative) were retrieved from the chart. The following demographic data were collected: age, weight, sex, the underlying disease process, preoperative pediatric end stage liver disease (PELD) score for patient under the age of 12 years, and model for end stage liver disease (MELD) score for patients over the age of 12 years. The following intraoperative details were documented: duration of operation, graft type (*living*,* reduced*,* or whole cadaver*) [6, 7], closure or nonclosure of the abdominal incision, and the volume of blood and blood products infused during the surgical procedure. The following postoperative data were collected: duration of ventilation, the PRISM III [8, 9] within 12 hours of admission to the PCCU, oxygenation index (OI = FiO_2_·MAP/PaO_2_), PaO_2_/FiO_2_ ratio during the first three days of ventilation, volume of blood products administered, the LOS in PCCU, and the 28 day mortality. The following complications were collected: pleural effusion, infection (culture proven), acute rejection, and intravascular thrombosis (confirmed by ultrasound and/or laparotomy). The data for the first three days of ventilation were collected from the ventilator flow sheet at approximately noontime (10^00^–14^00^) or the first data available after 6^AM^ when the former were missing. Acute rejection was defined taking into account clinical parameters during the first 100 days [10, 11]. A number of patients required more than one transplant. A transplant that took place after discharge from the PCCU and not less than 28 days after the previous transplant was considered as a separate transplant patient.

### 2.1. Statistical Analysis

Statistical analysis was performed using SPSS, version 18 (SPSS, Inc., Chicago IL). Normally distributed continuous variables were reported as means with standard deviations (SD), while skewed continuous variables were described as medians with interquartile range (IQR 25–75%). Categorical data were summarized using percentages. Spearman's rank correlation coefficient was used to assess the strength of the relationship between skewed continuous variables. The chi-square test or Fisher's exact test was used to compare differences in proportions for categorical variables. The independent samples* t*-test was used to compare mean differences in normally distributed continuous variables between two groups, and the Mann-Whitney* U* test was used to compare skewed continuous variables between two groups. The one-way ANOVA or Kruskal Wallis test was used to compare differences in the means and medians between three groups, respectively. We also computed multiple regression models with days of ventilation and ICU days as the dependent variables. Only those variables that were significantly associated with the outcomes in the univariate analysis were included as predictor variables in the regression. A *P* value of <0.05 was considered statistically significant.

## 3. Results 

### 3.1. Baseline Characteristics

A total of 151 patients who received 161 liver transplants during 1988–2011 were identified. Six patients were excluded because of missing data or because they received bowel and liver transplant. A total of 149 transplants in 145 patients were analyzed. Eight patients underwent more than one liver transplant; 6 retransplants took place within the same admission to the PCCU. The others (*n* = 4) took place within 38–810 days. The main reasons for retransplant were hepatic artery thrombosis and chronic rejection. The demographics are shown in Table 1.

### 3.2. Length of Stay and Length of Mechanical Ventilation

The median length of stay in the PCCU was 7 days (4.0–12.0). The median length of MV was 3 days (1.0–6.2). The median length of stay in the PCCU after extubation was 3 days (2.0–5.0) days.Seventeen patients (11%) were unable to have their incision closed. They were significantly younger, with a median age of 0.9 yrs (0.6–1.4) compared to a median of 3 yrs (1.0–7.7) (*P* < 0.001). It required a median of 5.5 (4.5–8.5) days after surgery to close the incision. These patients stayed a median of 12 days (9.0–18.2) in the PCCU and received a median of 8 days (6.0–11.5) of MV compared to a median of 6 days (4.0–10.5) (*P* < 0.01) and 2 days (1.0–6.0) (*P* < 0.001), respectively, in the other patients.

A multiple regression analysis for the patients who had their incision closed (close abdomen) was performed (Table 2) for all variables that were identified to correlate with the outcomes (length of mechanical ventilation or length of stay in the PCCU) in the univariate analysis.

### 3.3. Critical Care Course

The list of complications examined is shown in Table 3. Only one patient with a pleural effusion received a chest tube for drainage. Forty one patients (28%) had 49 infections. The most frequent source of infection was bacteremia (26/49, 53%), lungs (12/49, 24%), and abdomen (11/49, 22%). The most frequent pathogens were bacteria (79%), candida (18%), and viruses (8%). The most frequent bacteria isolated were* Staphylococcus aureus*,* Enterococcus faecalis*, and* Pseudomonas aeruginosa*.

### 3.4. Comparison betweeen Old Group (1988–1999) and Recent Group (2000–2011)

As shown in Table 4, there were significant differences between the* old* and* recent* group.

## 4. Discussion

This study describes the early postoperative course after OLT in children in a single center. The main indication for OLT was extrahepatic biliary atresia (41%), which is similar to other reports in children [1, 12]. The majority (63%) of liver donors were cadaveric and almost half were reduced size. The use of a living donor (12%) became more common in the last two decades (Table 1) as shown by others [13]. In 22 years of OLT in children 27% took place in infants (<12 months of age) and less than 1% in neonates (<2 month). The early mortality rate within the first 28 days was 8%, consistent with other data provided in the literature [14]. Mechanical ventilation after OLT is associated with significant complications [15, 16], therefore, in the last decade a number of studies in adults [17] and children [18] have advocated for early or even immediate extubation in the operating room [17, 19, 20]. In contrast, in our institution the length of MV has increased in recent years (Table 4). This may be explained by the larger proportion of patients in whom the abdominal incision could not be closed at the time of surgery in the* recent* period (20%) compared to* old *period (5.6%) (Table 4) and to the younger age of patients in the* recent* group. The case series that describes early extubation in children post-OLT were mainly but not only in older children [21]. Furthermore early extubation involves different anesthetic techniques which were not used in our patients [21].

Pleural effusion was very common (28%) and right side was significantly more frequently involved. This is probably the result of a reaction to the trauma induced by retraction during surgery and placement of a foreign body in the subphrenic space, with transudation of ascitic fluid across the diaphragm [22]. Except in one case in which a chest tube was inserted, these effusions resolved spontaneously and did not affect the length of MV or LOS. Right hemidiaphragm paralysis secondary to a right phrenic nerve injury has been described but was not observed in any of our patients [5].

The major changes that took place in the recent years such as younger age of patients and more living donors (Table 4) followed the trends noted in other centers [23, 24] and likely reflect improvement in surgical techniques. Other major changes such as reduce infections, early rejection rates, and transfusion threshold were not reported previously.

Recent adult and pediatric literature suggests that RBC transfusions in the critically ill pose multiple risks [25] and restrictive transfusion practices with subnormal levels of Hgb are well tolerated by the critically ill and are associated with improved patient outcome when compared to a liberal transfusion strategy [26]. Therefore, it is expected that median Hgb in the first three days after surgery was significantly lower in the recent group (93 g·L^−1^, 89–112) compared to the old group (108 g·L^−1^, 97–121) (Table 4).

The relatively high incidence of infection after OLT [27] is attributed to the underlying poor medical condition, the complexity of the surgical procedure, and immunosuppression. A total of 49 infections in 41 patients (28%) is lower than the 44−74% reported by others [14, 27, 28]. The incidence of infection decreased significantly over the years from 40% in the old group to 22% in the recent group (Table 4).

The incidence of early hepatic artery thrombosis in children ranges between 15–20% and the incidence of portal vein thrombosis is usually significantly lower [29]. Our study shows a higher rate of portal vein thrombosis (11%) compared to the hepatic artery (10%). This is similar to the incidence reported by Ganschow et al. [14]. With improvement in surgical technique, fluid management, and anticoagulation monitoring and management, we expected a reduction in the incidence of vascular complications. We speculate that this was not observed in our study because in recent period patients were significantly younger (Table 4) with smaller vessels.

The prediction of LOS and length of MV after OLT has been examined in several studies in children and adults [16, 30]. In addition to the obvious increase in LOS and MV in patient whose incision could not be closed at time of surgery, a multiple regression analysis shows that LOS is affected by age and OI on the second day after surgery. Length of MV is affected by age, amount of blood administered during surgery, PRISM score, and vascular complication.

### 4.1. Study Limitations

This study has several limitations. It is a single center study with relatively small number of OLT. It is a retrospective study with the usual limitations associated with this type of study. Certain complications (e.g., bleeding) were not assessed because of the difficulty in assessing them in a retrospective study.

## 5. Conclusion 

The course after OLT in the PCCU is relatively long and is associated with a relatively high rate of complications. In recent years OLT was performed in significantly younger children; living donors were more common, the transfusion threshold increased, rate of postoperative infection, and suspected rejection was reduced; However patients required significantly longer period of MV and a longer LOS in the PCCU.

## Figures and Tables

**Table 1:** Demographics of 145 patients who underwent 149 transplants.

Age (y)	4.5 ± 4.3, 2.7 (0.9–7)^a^
Weight (kg)	16.8 ± 12.7, 11.9 (18.0–20.5)
Male (%)	80 (54)
Cadaver (%)∗	98 (63)
Reduced cadaver (%)∗	39 (25)
Living donor (%)∗	18 (12)
PRISM on admission	8.0 ± 5.6, 7 (4–12)
PELD	15.0 ± 12.5, 15 (6–24)
MELD (13 patients >12 y)	59.9 ± 25.2, 66 (59–797)
Etiology	
Biliary atresia	61 (41)
Cholestasis	20 (13)
Fulminate hepatitis	10 (7)
Metabolic disease	10 (7)
Idiopathic cirrhosis	7 (5)
Hepatoblastoma	7 (5)
Others^b^	34 (23)

^a^Median with the interquartile; ^b^primary and acquired etiology; ∗out of 155 transplants.

**Table 2:** Multiple regression analysis assessing the relative effect of predictive variables for length of ventilation and length of stay in the PCCU.

Prediction variables	Length of mechanical ventilation		Length of stay in the PCCU	
	*B*	Beta	*B*	Beta
Age	−0.2∗	−0.278	−0.203∗	−0.256
PELD	0.02	0.086	—	—
Blood trasfused in OR	0.166∗	0.293	−0.054	−0.061
PRISM	0.036∗	0.202	0.031	0.168
Infection	0.188	0.170	—	—
PaO_2_/FiO_2_ first day	0.000	−0.085	0.000	−0.036
OI second day	—	—	0.436∗∗	0.396
Pleural effusion	0.162	0.095	—	—
Suspected rejection	−0.078	−0.064	—	—
Thrombosis	0.533∗	0.221	—	—
Creatinine postop	0.014	0.008	—	—

OR, operation room; OI, oxygenation index, *P < 0.05; **P < 0.001.

**Table 3:** Clinical profile and complications of 149 transplants (%).

Acute rejection	36 (24)
Infection	49∗
Bacteremia	26
Thromboembolism	
Hepatic artery	9 (6)
Portal vein	11 (7)
Both	4 (3)
Pleural effusion	
Right	35 (23)
Left	3 (2)
Bilateral	5 (3)
Effusion with chest tube	1 (0.7)
Mortality	12 (8)

*49 infections in 41 transplants, 7 patients had more than one infection.

**Table 4:** Comparison of transplants before (old group) and after year 2000 (recent group) period.

	1988–1999 (*n* = 90)	2000–2011 (*n* = 59)	*P*
Age (months)	40.5 (15–100)	17.0 (9.0–50.2)	0.004
Weight (kg)	12.9 (9.4–21.6)	10.0 (7.6–18.5)	0.07
<10 kg	28.0 (30)	26.0 (43)	0.14
Living donor	5.0 (5.6)	13.0 (22)	0.005
Open abdomen (%)	5.0 (5.6)	12.0 (20)	0.001
Median PELD	14 (2–21)	15 (9–27)	0.087
Median PRISM	6.0 (4–10)	10.0 (4–13.2)	0.014
Etiology			
Biliary atresia (%)	31 (34)	30 (51)	0.07
Cholestasis (%)	15 (17)	5 (8)	0.2
Fulminant hepatitis (%)	8 (9)	2 (3)	0.3
Metabolic disease (%)	4 (4)	6 (10)	0.3
Hepatoblastoma (%)	2 (2)	5 (8)	0.02
Median OR time (min)	458 (398–555)	520 (520–652)	0.047
Median LOS (days)	6.0 (4–9)	8.5 (6–17)	<0.001
Ventilation (days)	2.0 (1–5)	5.0 (2–10)	<0.001
Ventilation (close incision)	2.0 (1–4)	4.0 (2–7)	0.003
OR blood (mL/kg)	31.0 (18–60)	54.0 (28–116)	0.002
Median first 3 days Hb (g*·*L^−1^)	108 (97–121)	93.0 (89–112)	0.007
Infection (%)	36.0 (40)	13.0 (22)	0.04
Bactermia (%)	19.0 (21)	7.0 (12)	0.02
Thrombosis (%)	13.0 (14)	11.0 (19)	0.6
Suspected rejection (%)	30.0 (33)	6.0 (10)	0.002
Mortality (28 days)	6.0 (6.6)	6.0 (10.2)	0.6
